# Relationship between characteristics of spinopelvic alignment and quality of life in Japanese patients with ankylosing spondylitis: a cross-sectional study

**DOI:** 10.1186/s12891-020-3040-z

**Published:** 2020-01-18

**Authors:** Tatsuya Sato, Ikuho Yonezawa, Hisashi Inoue, Kurisu Tada, Shigeto Kobayashi, Eri Hayashi, Naoto Tamura, Kazuo Kaneko, Juntendo AS Research Group

**Affiliations:** 1 Department of Orthopedic surgery, Juntendo University School of Medicine, Tokyo, Japan; 2 Department of Orthopaedic Surgery, Sangubashi Spine Surgery Hospital, Tokyo, Japan; 3 Department of Internal Medicine and Rheumatology, Juntendo University School of Medicine, Tokyo, Japan; 4 Department of Internal Medicine (Rheumatology), Juntendo Koshigaya Hospital, Saitama, Japan

**Keywords:** Ankylosing spondylitis, Health-related quality of life, Spinopelvic alignment, Degenerative lumbar kyphoscoliosis

## Abstract

**Background:**

Studies on characteristic spinal deformities in Japanese patients with ankylosing spondylitis (AS) and data demonstrating a relationship between health-related quality of life (HRQOL) and spinopelvic alignment in these patients are lacking.

**Methods:**

In this cross-sectional study, 50 patients with AS and without a surgical history, vertebral body fracture, or scoliosis as well as 30 control patients with degenerative lumbar kyphoscoliosis (DLKS) were included. Data collected included patient sex, age, spinopelvic parameters on sagittal full-spine standing radiographs, and HRQOL questionnaire responses. Student’s *t*-test was used to compare the characteristics of spinopelvic parameters between the groups. A multiple regression analysis was performed to analyze correlations between spinopelvic parameters and HRQOL in the AS group.

**Results:**

Global kyphosis (GK; T1–12 angle) was significantly greater in the AS group than in the DLKS group (*P* < 0.001), whereas the pelvic tilt (PT; posterior PT angle) was smaller in the AS group (*P* = 0.006). Radiographic parameters correlated with HRQOL in the AS group. Multiple regression analysis identified the sagittal vertical axis (SVA) and sacral slope (SS) as factors influencing the SRS-22 total score and SVA and GK as factors influencing Japanese Orthopaedic Association Back Pain Evaluation Questionnaire mental health (subdomain).

**Conclusions:**

Patients with AS did not use lumbar lordosis or posterior PT to compensate for their large thoracic kyphosis due to spinopelvic ankylosis. These patients showed a unique compensation pattern. The correlation/regression analysis revealed a correlation between radiographic parameters and HRQOL in patients with AS, with particular importance of SVA, SS, and GK for clinical results in AS.

## Background

Ankylosing spondylitis (AS) is a seronegative spondyloarthropathy that causes sacroiliitis, spondylitis, and thoracic kyphosis [1]. AS has been reported to be strongly associated with serum HLA-B27. The HLA-B27–positive rate and AS prevalence vary greatly between ethnicities, with both rates high in European and American whites and Taiwanese citizens whereas they are extremely low in Japanese citizens. The severity of AS progression is characterized by compromised sagittal balance due to thoracic kyphosis, ankylosis of the sacroiliac and hip joints, and a limitation of chest wall expansion [2]; however, the association between AS progression and health-related quality of life (HRQOL) remains unknown. In the only previous report dealing with the association between sagittal balance and HRQOL in AS, the participants were Korean and had near-normal thoracic kyphosis [3]. Although many studies have been published on the usefulness of surgical treatment for AS and its major complications [4], to our knowledge, no study has described the relationship between sagittal balance and HRQOL, which is important before the disease has worsened enough to indicate surgical intervention. Currently, the Bath AS Metrology Index is sometimes used as a measure to assess spine/hip joint mobility and limb postures in AS [5], but it does not fully reflect the sagittal balance. In addition, many studies have documented associations between sagittal balance and HRQOL for grading spinal deformity progression in adults [6]. However, the relationship between sagittal balance and HRQOL in AS remains unclear. Our aims were (i) to characterize the sagittal spinal deformity associated with progression of thoracic kyphosis in AS by comparing sagittal spinopelvic parameters between AS and degenerative lumbar kyphosis (DLKS) and (ii) to reveal the relationship between sagittal spinopelvic parameters and HRQOL in AS and to identify sagittal spinopelvic parameters that affect HRQOL in patients with AS.

## Methods

We studied 50 patients (42 men, eight women; average age, 44.3 ± 14.3 years) with AS from August 2015 to November 2016. All patients, Japanese, met the most recent modified New York criteria for the diagnosis of AS [7]. The inclusion criteria were as follows: (1) patients with a T1–12 angle (global kyphosis: GK) < 120°; (2) absence of scoliosis or with a coronal curve < 15°; (3) absence of previous spinal surgery, pseudarthrosis, spinal fracture, or discitis; and (4) Ankylosing Spondylitis Disease Activity Score [ASDAS] < 3.5 [8]. Another DLKS group was also enrolled for comparison of sagittal spinopelvic parameters. There were five men and 25 women (average age, 68.4 ± 12.8 years). The inclusion criteria were as follows: (1) patients categorized as having a sagittal balance defect for sagittal modifiers according to the adult spinal deformity classification by Schwab et al. [9] and associated back pain as a chief complaint; and (2) patients with no history of fresh vertebral body fracture within 3 months, any neurological deficit, or spinal surgery. For both groups, HRQOL measures included scores on the Oswestry Disability Index (ODI) questionnaire, Roland–Morris Disability Questionnaire (RDQ), Scoliosis Research Society Questionnaire (SRS-22; total and four subdomains: activity, appearance, mental, and satisfaction), and Japanese Orthopaedic Association Back Pain Evaluation Questionnaire (JOABPEQ) [10] (five subdomains: pain-related disorders, lumbar spine dysfunction, gait disturbance, social life dysfunction, and psychological disorders). We examined only spinal column-related pain by asking patients to indicate the site of pain [11] and excluded pain in the buttocks, hips, knees, and shoulder joints. This study was approved by the medical ethics committee of Juntendo University Hospital (17–015).

Standing posteroanterior and lateral radiographs of the entire spine were obtained from patients with AS and DLKS in the fist-on-clavicle position. All radiographs were acquired in digital format. Using Surgimap (version 2.2.2; Spine Software, New York, NY), parameters related to sagittal spinopelvic alignment were then measured by the same spine surgeon.

**Fig. 1:**
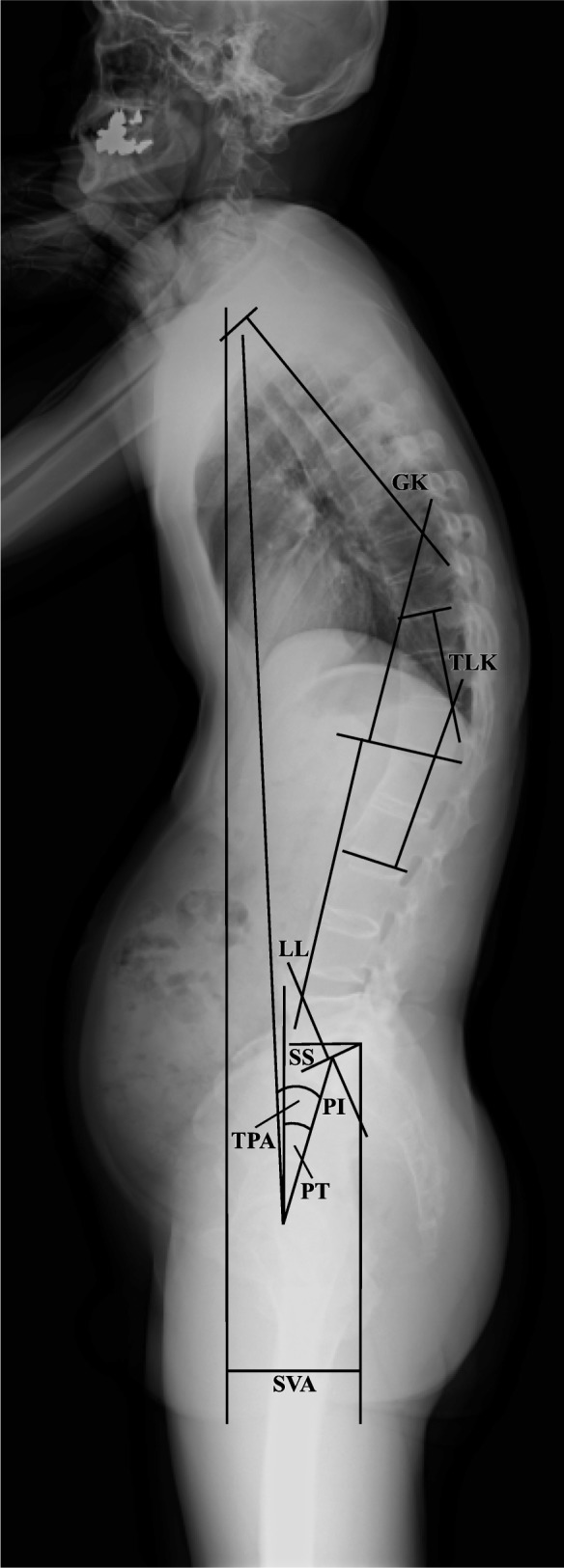
Sagittal spinopelvic parameters for radiologic measurements. The angle between the sacral endplate and the horizontal was defined as sacral slope (SS), the angle between the line joining the center of the sacral endplate and hip axis and the vertical axis was defined as pelvic tilt (PT), and the angle between a line perpendicular to the sacral endplate and a line joining the center of the sacral plate and hip axis was defined as pelvic incidence (PI). The following three measures were evaluated using Cobb’s method: global kyphosis (GK) was measured as the angle between the upper endplate of the T1 vertebra and the lower endplate of the T12 vertebra; thoracolumbar kyphosis (TLK) was measured as the angle between the upper endplate of the T10 vertebra and the lower endplate of the L2 vertebra; and lumbar lordosis (LL) was measured as the angle between the upper endplate of the L1 vertebra and the lower endplate of the L5 vertebra. The horizontal distance of a C7 plumb line dropped from the C7 body center to the posterosuperior corner of the S1 body was defined as sagittal vertical axis (SVA). Anterior displacement of the sagittal plumb line was considered as positive. The angle between the line from the femoral head axis to T1 body center and the line from the femoral head axis to the center of the S1 superior endplate was defined as T1 pelvic angle (TPA)

The following radiographic parameters were considered: sacral slope (SS), pelvic tilt (PT), pelvic incidence (PI), GK, thoracolumbar kyphosis (TLK), lumbar lordosis (LL), sagittal vertical axis (SVA), and T1 pelvic angle (TPA) (Fig. 1). As an evaluation method combining the effects of anterior truncal tilt and retroversion of the pelvis, TPA is a parameter that is reportedly resistant to effects from knee flexion and pelvic retroversion [12].

Statistical analyses were performed using SPSS 21 statistical software (SPSS, Inc., Chicago, IL, USA). Statistical data are presented as mean  ±  SD. In our study, the two groups were compared using Student’s *t*-test, and correlation analyses also were performed using Pearson’s correlation to demonstrate the relationship between variables. Furthermore, to identify parameters that accurately correlate with the clinical outcome, we performed multiple regression analyses. Differences were regarded as significant when the *P* value was  <  0.05.

## Results

The geometric parameters of sagittal spinal and pelvic alignment in the AS and DLKS groups are listed in Table 1. When comparing the AS and DLKS groups, GK was significantly greater (45.9 ± 17.8 vs. 30.3 ± 15.7, *P* < 0.001) and PT was smaller (28.7 ± 12.4 vs. 35.3 ± 8.31, *P* = 0.006) in the former group, whereas no significant differences were found in SVA (85.8 ± 75.3 vs. 86.1 ± 57.1, *P* = 0.986), SS (23.2 ± 12.2 vs. 24.4 ± 14.6, *P* = 0.707), PI (51.3 ± 11.1 vs. 54.8 ± 13.2, *P* = 0.208), or LL (28.5 ± 20.7 vs. 24.9 ± 16.0, *P* = 0.407). Table 2 shows correlations among age, BMI, and sagittal spinopelvic parameters in patients with AS.

**Table 1:** Comparison of sagittal spinopelvic parameters between the groups

	AS (*n* = 50)	Range	DLKS (*n* = 30)	Range	*P* value
Gender (male/female)	42/8		5/25		< 0.001**
Age	44.3 ± 14.6	20 to 75	68.5 ± 12.8	27 to 84	< 0.001**
Body mass index (BMI)	23.9 ± 4.18	15 to 36	22.0 ± 3.61	17 to 29	0.045*
Sagittal vertical axis (SVA)	85.8 ± 75.3	− 38 to 282	86.1 ± 57.1	− 58 to 196	0.986
T1 pelvic angle (TPA)	28.3 ± 15.9	− 1.0 to 67	35.0 ± 9.18	19 to 56	0.02*
Sacral slope (SS)	23.2 ± 12.2	3 to 52	24.4 ± 14.6	−5 to 63	0.707
Pelvic tilt (PT)	28.7 ± 12.4	5 to 58	35.0 ± 8.31	24 to 53	0.006**
Pelvic incidence (PI)	51.3 ± 11.1	34 to 74	54.8 ± 13.2	30 to 80	0.208
Global kyphosis (GK)	45.9 ± 17.8	−18 to 79	30.3 ± 15.7	1 to 57	< 0.001**
Thoracolumber kyphosis (TLK)	16.4 ± 11.6	1 to 52	25.7 ± 18.7	−5 to 69	0.019*
Lumbar lordosis (LL)	28.5 ± 20.7	−18 to 79	24.9 ± 16.0	1 to 57	0.407

**Table 2:** The correlation coefficient between age, BMI, and sagittal spinopelvic parameters for patients with AS

Correlation coefficient (r) between sagittal spine parameters and pelvic measures for patients with AS	Age	BMI	SVA	TPA	SS	PT	PI	GK	TLK	LL
Age		0.153	0.129	0.175	− 0.337*	0.323*	0.005	0.367*	0.446*	− 0.148
Body mass index (BMI)			0.162	0.225	−0.197	0.233	0.066	0.376*	0.265	− 0.088
Sagittal vertical axis (SVA)				0.857*	−0.462	0.665*	0.208	0.138	0.353*	− 0.720*
T1 pelvic angle (TPA)					−0.618*	0.899*	0.287	0.228	0.412*	−0.750*
Sacral slope (SS)						−0.594	0.420	−0.198	−0.563*	0.843*
Pelvic tilt (PT)							−0.608*	0.316*	0.407*	0.420*
Pelvic incidence (PI)								0.118	−0.191	0.246
Global kyphosis (GK)									0.512*	0.094
Thoracolumbar kyphosis (TLK)										−0.403*
Lumbar lordosis (LL)										

The correlations between sagittal spinopelvic parameters and HRQOL questionnaires for patients with AS are shown in Table 3. Among the HRQOL questionnaires, ODI, SRS-22 total, SRS-22 appearance, JOABPEQ social life dysfunction, and JOABPEQ psychological disorder scores correlated statistically with each parameter. The statistical correlations between HRQOL and age, BMI, and PI tended to be insignificant.

**Table 3:** The correlation coefficient between sagittal spinopelvic parameters and HRQOL questionnaires for patients with AS

	ODI	SRS-22		JOABPEQ		ASDAS
		Total	Appearance	Social life dysfunction	Psychological disorders	
Age	0.081	−0.208	−0.304*	− 0.028	− 0.090	0.195
Body mass index (BMI)	0.112	−0.089	−0.210	−0.071	− 0.065	0.093
Sagittal vertical axis (SVA)	0.347*	−0.467*	−0.569*	−0.374*	−0.461*	0.276
T1 pelvic angle (TPA)	0.258	−0.441*	−0.597*	−0.269	−0.436*	0.232
Sacral slope (SS)	−0.392*	0.512*	0.631*	0.282*	0.423*	−0.295*
Pelvic tilt (PT)	0.218	−0.425*	−0.581*	−0.229	−0.386*	0.249
Pelvic incidence (PI)	−0.190	0.106	0.067	0.044	0.019	−0.063
Global kyphosis (GK)	0.275	−0.345*	−0.281*	−0.268	−0.396*	0.221
Thoracolumbar kyphosis (TLK)	0.300*	−0.298*	−0.411*	−0.224	−0.209	0.082
Lumbar lordosis (LL)	−0.337*	0.452*	0.572*	0.249	0.368*	−0.228

The results of multiple regression analyses on sagittal spinopelvic parameters and clinical outcomes for patients with AS are shown in Table 4. SVA and SS were significantly correlated with SRS-22 total scores and SRS-22 appearance scores. SVA and GK were significantly correlated with JOABPEQ social life dysfunction.

**Table 4:** Multiple regression analysis in patients with AS

Variables	Coefficient	*t*	*P* value
SRS-22 total			
Sagittal vertical axis (SVA)	−0.293	−2.172	0.035
Sacral slope (SS)	0.376	2.794	0.008
SRS-22 appearance			
Sagittal vertical axis	−0.353	−3.022	0.004
Sacral slope	−0.468	4.009	< 0.001
JOABPEQ social life dysfunction			
Sagittal vertical axis	−0.414	−0.342	0.001
Global kyphosis (GK)	−0.339	−2.805	0.007

## Discussion

In adults with spinal deformity, sagittal balance has been reported to be related closely to HRQOL, and sagittal spinopelvic parameters are known to be important factors for treatment decisions [6]. However, spinal deformity in AS is a pathological condition that is different from adult spinal deformity, and the characteristics of sagittal balance and its relationship with HRQOL are largely unknown in patients with AS. In recent years, the importance of corrective treatment for AS spinal deformity has been increasingly acknowledged, and many studies have reported associations between sagittal spinopelvic parameters and HRQOL for the objective evaluation of surgical correction of AS spinal deformity [13]. However, studies on sagittal spinopelvic parameters and HRQOL in nonsurgical treatment for AS spinal deformity are scarce [3].

In recent years, interracial differences have been reported for the association between sagittal alignment of the spinal column and HRQOL in adult spinal deformity [14]. These differences suggest that the pathological evaluation of sagittal balance of the spinal column should be performed depending on the ethnicities of patients, not only in adults with vertebral deformity but also in those with AS. In particular, AS prevalence rates vary greatly among ethnicities, with rates ranging from 0.52% in the United States [15] and 0.19 to 0.54% in Taiwan [16] to 0.0065% in Japan [17]. This disparity is another justification for separate analyses by ethnicity.

In the sagittal balance comparison of AS and DLKS in this study, each gender ratio was different. This is because AS often occurs in males [17], and DLKS often occurs in females [18], as the respective disease characteristics. Although male AS patients show radiological progression, including the development of syndesmophytes [19], the effects of gender difference on sagittal balance have not been clarified. Furthermore, in healthy adults, it has been reported that females in their 70s have larger PTs and PIs than males [20], and spinopelvic parameters has been reported to be similar for males and females in their 30s [21]. However, there is no large-scale study on the impact of gender differences on sagittal balance, including the adult spinal deformity classification by Schwab et al. [9], and this is unclear in DLKS as well as in AS.

Sagittal balance of the spinal column and pelvic morphology in patients with AS are reported to be different from those in healthy individuals [1, 22]. Previous studies have compared the characteristics of sagittal alignment in patients with AS and healthy individuals [3, 4, 23], but to our knowledge, no comparisons have been made with DLKS. Given a comparison was being made with patients with DLKS, we were able to analyze the compensatory function of the sagittal alignment in patients with AS.

In this study, an AS population with relatively large thoracic kyphosis was compared with a DLKS population. The results showed that, despite a similar SVA in the two populations, the former had significantly greater thoracic kyphosis and significantly smaller posterior PT with no significant difference in LL. In addition, although SVA was equivalent, TPA [12], which combines SVA and PT information, was significantly smaller in patients with AS. This finding demonstrated a sagittal alignment characteristic of AS, suggesting that the effect of compensation by posterior PT, which is usually found in patients with DLKS, is small. One study presumed that pelvic retroversion restrictions (i.e., small PT angles) in AS patients are sagittal malalignment corrections by flexion of the knees and/or plantar flexion of the ankles [22] . However, since this mechanism cannot be substantiated by this study, further investigations involving the lower limbs are necessary.

Of the HRQOL measures, ODI, SRS-22 total, SRS-22 appearance, JOABPEQ social life dysfunction, and JOABPEQ psychological disorder scores showed statistical correlations with sagittal spinopelvic parameters in patients with AS. This result appears to indicate that compromised sagittal balance of the spinal column decreases the HRQOL of these patients.

A study on Korean patients with AS and near-normal and early-stage kyphosis reported that SVA, SS, and LL showed an important correlation with HRQOL for patients with AS [3]. A study on associations between HRQOL and SVA, TPA, spinosacral angle, and spinopelvic angle (SPA) in patients with AS and with a relatively large kyphosis has shown that SPA was significantly correlated with ODI [24]. In our study, the questionnaires most relevant for AS evaluation were the SRS-22 and JOABPEQ. Furthermore, the HRQOL was significantly correlated with SVA, SS, and GK. AS progression has been reported to be characterized by thoracic kyphosis and ankylosis [6]. In our participants who were likely to have relatively advanced thoracic kyphosis and ankylosis, GK was newly identified as an important sagittal spinopelvic parameter for AS evaluation in addition to SVA and SS.

Our study revealed the presence of a sagittal balance mechanism unique to patients with AS. In addition, thoracic kyphosis associated with AS progression was shown to be closely related to lower back pain, and its degree was closely related to SRS-22 and JOABPEQ scores. Furthermore, we identified SVA, SS, and GK as important sagittal spinopelvic parameters related to HRQOL.

This study has some limitations. First, the effects of coronary imbalance were not considered although patients with kyphoscoliosis were included as the control group. Second, because patients with kyphosis without scoliosis are scarce, the AS and DLKS groups were not precisely matched in age. Third, the effects of inflammation itself on HRQOL cannot be ruled out. Fourth, it was difficult to discuss possible effects of medical treatment and deformity correction surgery intervention because this was a cross-sectional study.

## Conclusions

The findings of this study demonstrated that sagittal balance was clearly different between patients with AS and DLKS, and that those with AS assumed a characteristic posture to compensate for the forward movement of the central axis (C7 plumb line). Furthermore, SRS-22 and JOABPEQ scores correlated with HRQOL in patients with AS, and SVA, SS, and GK were significant sagittal spinopelvic parameters that correlated with HRQOL.

## Data Availability

The datasets used and/or analyzed during the present study are available from the corresponding author on reasonable request.

## Abbreviations

AS: Ankylosing spondylitis
ASDAS: Ankylosing Spondylitis Disease Activity Score
DLKS: Degenerative lumbar kyphoscoliosis
GK: Global kyphosis (T1–12 angle)
HRQOL: Health-related quality of life
JOABPEQ: Japanese Orthopaedic Association Back Pain Evaluation Questionnaire
LL: Lumbar lordosis
ODI: Oswestry Disability Index
PI: Pelvic incidence
PT: Pelvic tilt
RDQ: Roland–Morris Disability Questionnaire
SPA: Spinopelvic angle
SRS-22: Scoliosis Research Society Questionnaire
SS: Sacral slope
SVA: Sagittal vertical axis
TLK: Thoracolumbar kyphosis
TPA: T1 pelvic angle
